# A Technological Understanding of Biofilm Detection Techniques: A Review

**DOI:** 10.3390/ma13143147

**Published:** 2020-07-15

**Authors:** Spyridon Achinas, Stijn Keimpe Yska, Nikolaos Charalampogiannis, Janneke Krooneman, Gerrit Jan Willem Euverink

**Affiliations:** 1 Faculty of Science and Engineering, University of Groningen, 9747 AG Groningen, The Netherlands; s.k.yska@student.rug.nl (S.K.Y.); j.krooneman@rug.nl (J.K.); g.j.w.euverink@rug.nl (G.J.W.E.); 2 Department of Urology, SLK Kliniken am Gesundbrunnen, 74078 Heilbronn, Germany; n.charalampog@outlook.com

**Keywords:** biofilm, adhesion, detection techniques, materials, bioreactors

## Abstract

Biofouling is a persistent problem in almost any water-based application in several industries. To eradicate biofouling-related problems in bioreactors, the detection of biofilms is necessary. The current literature does not provide clear supportive information on selecting biofilm detection techniques that can be applied to detect biofouling within bioreactors. Therefore, this research aims to review all available biofilm detection techniques and analyze their characteristic properties to provide a comparative assessment that researchers can use to find a suitable biofilm detection technique to investigate their biofilms. In addition, it discusses the confluence of common bioreactor fabrication materials in biofilm formation.

## 1. Introduction

Bioreactors are used in many biotechnological applications from laboratory experiments to large-scale production processes [1,2,3]. Conventional materials in the fabrication of bioreactors are stainless steel and glass [4]. These materials are expensive, and therefore, production processes that use bioreactors are considered pricey [5]. Recently, much research has been dedicated to finding alternative construction materials for bioreactors [4,5]. One possible replacement for conventional materials applicable for the construction of bioreactors is polymer resin. Previous research has led to the construction of a 3D-printed anaerobic bioreactor constructed of resin as a cheaper alternative for conventional steel and glass bioreactors. Although resin is a relatively inexpensive construction material for bioreactors, it has with the same problem as conventional bioreactor construction materials, which is biofouling.

Biofouling can be referred to as the “unwanted” deposition and growth of biofilms [6], where biofilms are organized aggregates of microorganisms living within an extracellular polymeric substance (EPS) matrix that they produce [7]. The biggest issue concerning biofouling is that the microorganisms that cause biofouling can survive, even when 99.9% are removed from the feed stream, they adapt their growth rate, multiply, and relocate [8]. The large variety of problems caused by biofouling can be separated into two categories: (1) the formation of biofilm can result in health problems by the liberation of cell clusters out of the EPS matrix [7,9] and (2) the formation of biofouling on process equipment and open surfaces can result in a reduction in efficiency [10]. Biofouling is a persistent problem in almost any water-based application [6]. Apart from the problems caused by biofouling in bioreactors, biofouling also causes problems that affect many other industries. Examples of such industries are the dairy industry, biodiesel production industry, stem cell cultivation industry, shipping industry, surgical implants industry, and laboratories [1,3,11,12,13].

To irradiate biofouling causes problems in bioreactors and other industries, so monitoring systems are necessary to develop efficient anti-biofouling strategies [14]. These monitoring systems can be referred to as biofilm detection techniques [9,14]. Due to the wide variety of industries affected by the problem of biofouling, many different biofilm detection techniques have been developed throughout the past decades [9]. Two examples of such detection techniques and their application are (1) the visible and near-infrared processing technique (V&NIR), which is used to detect biofilm on monuments [15] and is based upon the properties of biological material to differently absorb and reflect light in different spectral bands [15], and (2) the cumulative sum (CUSUM) control chart, which detects biofilms within heat exchangers by the slope change of the heat transfer resistance value (Rf) [16]. These are two examples of detection techniques that have not been applied to detect biofilms in bioreactors; however, there are also detection techniques that have been applied in bioreactors. An example of such a detection technique is confocal laser scanning microscopy (CLSM), which uses a specially installed beam splitter to detect the light reflected from all objects [17].

Notably, the literature supporting the different biofilm detection techniques has not focused on the applicability of the techniques with regard to biofilm detection in bioreactors. Due to the dearth of information regarding biofilms in bioreactors, this report aims to provide a review of all the different biofilm detection techniques, their various properties, and results, and employed this overview as a selection tool that is capable of comparing the different selection techniques and will result in one or more options to apply to bioreactors. Although the focus of this report is in the application of biofilm detection techniques for bioreactors, all biofilm detection techniques must be taken into consideration, and therefore the literature that applies detection techniques, not for bioreactors but elsewhere, must also be considered. Thus, the results of this research will not lead to a strategy to overcome or reduce biofouling, but will provide a technological guide that assists the biofilm-related research in bioreactors. 

## 2. Theoretical Facets of Biofouling

### 2.1. Biofouling Phenomenon

**Figure 1:**
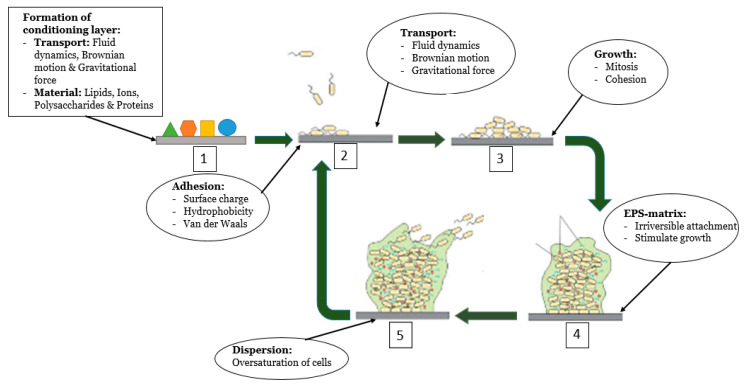
A visualization of the five phenomena of biofilm formation. The figure indicates the conditions of the medium and the specific surface on which microbial adhesion occurs; the initial adherence of microorganisms to the surface; the proliferation (or else microcolony formation); and the maturation of the biofilm architecture with the presence of the polymeric matrix and its dispersion [7,9].

Biofouling is a sequential, four-step phenomenon governed by several physical, chemical, and biological factors: attachment, proliferation, maturation, and dispersion, as depicted in Figure 1 [7,18,19]. However, the literature does not focus on the formation of biofilm within bioreactors. Since biofouling affects many industries, biofouling occurs on many different surfaces. Therefore, prior to the adhesion of microorganisms to the surface, the properties of the surface that influence biofouling are added to the system. 

The first phenomenon that occurs is the formation of a conditioning layer or film on the surface. After contact between the fluid and surface, the surface is covered by organic and inorganic material present in the liquid [20]. The conditioning layer serves as the foundation on which a biofilm grows [10]. This layer is composed of many particles, both organic and inorganic (i.e., ions, proteins, polysaccharides, and lipids), that are present in the bulk fluid. These particles are transported to the surface using gravitational force, fluid dynamics, and Brownian motion [7,14]. The formation of a conditioning layer strongly affects the physical-chemical properties of the surface such as the surface charge (electrokinetics) and the hydrophobicity of the surface [6,15]. Hereafter, microorganisms are transported from the fluid to the conditioned surface. Equal to the transportation of organic and inorganic material to form the conditioning layer, the forces that cause the transportation of microorganisms to the conditioned surface are gravitational force, fluid dynamics, and Brownian motion [15]. However, due to the formation of the conditioning layer, the properties of the surface have changed and interactions between the surface and microorganisms occur [6]; this attachment between the cell and substrate is termed cohesion [10].

In general, multiple species of microorganisms such as bacteria, algae, protozoa, and fungi are present within a fluid. The different species are attracted or repelled by a surface through the electrokinetics and hydrophobic properties of a surface and the van der Waals forces [7,20]. After the formation of the first layer of microorganisms, the electrokinetics and hydrophobicity of the initial surface remain attractive to microorganisms, however, the presence of the microorganisms attached to the surface now contributes to the growth of the biofilm. The cell-to-cell attachment of different microorganisms is called cohesion [10]. Apart from the attachment of other microorganisms, the biofilm also grows by mitosis. Mitosis is enabled by the extraction of nutrients from the fluid and the specific structure of biofilms enables the transportation of these nutrients to the deeper layers of microorganisms [7]. During the period of growth, the microorganisms become irreversibly attached to the conditioned surface, stimulated by several chemical reactions such as oxidation and hydration [10]. After the irreversible attachment of the initial layer, a rapid increase in the cell population is observed. This rapid increase is caused by the EPS originating as a protective layer for the cells. The chemical reaction between the initial layer and the surface together with the formation of the EPS-matrix that anchors the cells to the surface is called irreversible attachment [7,10,20]. Finally, an oversaturation of cells within the EPS-matrix results in the dispersion of microorganisms into the fluid.

### 2.2. Detection Techniques

There is a lack of consensus of the most appropriate techniques to detect biofilms in bioreactors [9]. Azeredo et al. (2017) created an overview of several detection techniques and separated the different biofilm detection techniques into four categories: chemical, physical, microscopical, and biological [9]. Techniques were classified according to the following definitions:

- Physical: when the total biomass of the biofilm can be obtained from dry or wet weight measurements.
- Chemical: when it uses dyes or fluorochromes that can bind to or adsorb onto biofilm components.
- Microscopical: when an imaging modality is used to detect the formation of biofilm (i.e., whenever a microscope is used).
- Biological: when a technique uses the estimation of cell viability in measuring and detecting biofilm formation.

Apart from these four categories, biofilm detection techniques also have other properties that can be used to qualify and categorize them: on-line monitoring, in situ monitoring, real-time monitoring, and are non-destructive, representative, accurate, reproducible, and automatic [9,21]. 

Furthermore, the results obtained by the different biofilm detection techniques can also differ. Possible results obtained by different detection techniques are microbial activity, total cell counts, 2D distribution of bacteria in the biofilm, 3D structure of biofilm, and the ability to identify different components of biofilms [9,13]. 

### 2.3. Materials for Bioreactors Fabrication

Conventional materials for constructing bioreactors are stainless steel and glass [4]. The materials used to fabricate a bioreactor must be able to withstand certain conditions while running including clean-up and sterilization [4]. Previous studies have elaborated on the construction of bioreactors able to meet those conditions. An example of such a study is the construction of a 3D-printed bioreactor that was constructed out of clear FLGPCL02 proprietary resin [5]. This example represents the possibilities of construction materials for bioreactors. The construction materials of bioreactors vary and the formation of biofilm in their surface has to be taken into account [4,5]. The ability, rate, and extent of adherence of microorganisms on a surface depend on the specific properties of the material. 

## 3. Technological Substratum of Detection Techniques

### 3.1. Physical

#### 3.1.1. Cumulative Sum (CUSUM) Control Chart

Boullosa-Falces et al. (2019) monitored the evolution of biofouling adherence to the internal surface of a heat exchanger through the slope change of the heat transfer resistance value (R_f_), which is a widely used technique that has been validated in numerous studies, and CUSUM control graphs [16].The value of R_f_ depends on the effect of biofilm growth on the boundary layer of the fluid and the turbulence in the interface area. The R_f_ is measured and compared with its previously measured values; if the resistance to heat transfer increases, this indicates that a change in the internal environment of the heat exchanger has occurred [22]. Thus, R_f_ is monitored and when it decreases, it is known that biofouling has occurred. Boullosa-Falces et al. (2017) [23] plotted the different values of R_f_ in a graph and table and showed the decrease in R_f_ and in this way, visualized the results of applying this technique. Moreover, several other techniques to detect biofouling within heat exchangers have been investigated, examples of which are acoustic, x-rays, optical, and ultrasound. The negative aspects of these techniques are the costs associated with them [16]. Moreover, in earlier research by Boullosa-Falces et al. (2017), the CUSUM method was applied to marine diesel engines to detect fluctuations in parameters [23]. These varying applications of CUSUM (in a heat exchanger and marine diesel engine) suggests that this technique is applicable in different areas such as bioreactors. However, there is no literature supporting this claim. Moreover, it can be concluded that the application of CUSUM depends on a parameter that is affected by biofouling. The current study examined resin as a bioreactor fabrication material to perform anaerobic digestion [5]. An indicator of the process becoming unstable is the pH of the medium. A decrease in pH indicates the instability of the process. If CUSUM is to be applied for bioreactors, pH could be a reference variable to apply. 

#### 3.1.2. Visible and Near-Infrared (V&NIR) Image Processing

V&NIR is based on the properties of biological materials to differently absorb and reflect light in different spectral bands. Different kinds of biological objects, in this case, different types of biofilm, have different spectral characteristics. In the article by Griskin et al. (2017), the proposed method obtained several images in the V&NIR spectral bands using a digital photo camera [15]. Hereafter, these images were analyzed by comparing them with well-known groups of vegetation indexes such as the normalized difference vegetation index (NDVI) and the enhanced normalized difference vegetation index (ENDVI). Image processing is a widely used technique and its application varies. Examples of the application of this technique are soil moisture analysis, bacterial monitoring of drinking water sources, the food industry, and antibacterial activity of textile materials [24,25,26,27]. Moreover, there are techniques that use visible light to trigger a reaction of the biofilm in question. An example of such a technique is provided by Zhiqiang et al. (2019), who fabricated nitric oxide (NO)-releasing amphiphiles and applied this to the biofilm, which triggered a reaction, and in turn released NO when exposed to visible light [28]. However, the latter technique differs from V&NIR since its implementation requires the fabricated NO-releasing amphiphiles and a microscope to image the reaction.

The main obstacle for the application of this technique concerning bioreactors is the necessity of a database containing the different species of biofilms so that a valid differentiation between the different species of biofilm can be made. Due to the dearth of research on this technique, such a database does not exist. Moreover, the creation of such a database is a time-consuming process and therefore was not within the scope of this report. If a suitable database is created, V&NIR image processing could lead to the in situ, non-destructive, and real-time detection of critical spots and the different species of biofilm present in a bioreactor. 

#### 3.1.3. Electrochemical Impedance (EIM) Spectroscopy

Impedance measurements look for changes in the bulk resistance of the solution, usually with a two-electrode technique [29]. A more extensive explanation of EIM spectroscopy is provided by Azeredo et al. (2017); the principal of EIM spectroscopy lies in the detection of changes in the diffusion coefficient of a solution, which is recorded as an electrochemical reaction measured on the electrode [9]. This reaction depends on the local mass transfer coefficient and reduces with increasing biofilm thickness [30]. Bonetto et al. (2014) investigated the properties of EIM spectroscopy to serve as a method in differentiating between microorganisms [31], which resulted in different measurements for different microorganisms, thus, EIM spectroscopy can also function as a tool to distinguish different microorganisms in the same medium. Since EIM spectroscopy detects biofouling by utilizing sensors, this technique could also be applied for the detection of biofilm in bioreactors. Research by Bimakr et al. (2018) assessed the possible use of EIM spectroscopy to detect biofilm through graphite and stainless-steel sensors in pipes for water drinking systems [32]. They assessed various materials for sensor applications including noble metals, carbon, and titanium; however, these studies have not been performed in an aqueous environment, which is necessary if EIM spectroscopy is to be applied for bioreactors. The sensors that accompany EIM spectroscopy can be mounted on the inner surface of bioreactors so that the biofilm detection can be performed in situ, real-time, and in a non-destructive manner. However, due to limited information concerning the application of EIM spectroscopy to detect biofilm in bioreactors, it is unknown as to whether the attachment of the sensors to the inner surface of the bioreactor might disturb the process or cause other problems. Furthermore, this technique is not capable of obtaining information regarding critical spots for biofilm on the inner surface of the bioreactor, and as the result obtained by applying EIM spectroscopy will be in the form of a graph, this graph solely represents the formation of biofilm and its thickness on the sensors [33]. 

#### 3.1.4. Nuclear Magnetic Resonance (NMR) Imaging

All molecules consist of nuclei and all atomic nuclei with an uneven number of protons and neutrons carry angular momentum or spin, and therefore a magnetic moment [34]. If a sample with nuclear spins is placed inside a strong, external magnetic field, the interaction of the magnetic moment with the external magnetic field causes the nuclear spins to align and thus create a small magnetization vector within the sample [35]. This magnetization can be manipulated by the application of radiofrequency pulses of a given power and duration. After the application of such frequency pulses, the magnetization vector changes with a specific, so-called, Larmor frequency. Nuclear magnetic resonance (NMR) imaging is based upon the fact that the Larmor frequency is proportional to the polarizing magnetic field. Given this proportionality, 2D- and 3D-images of the spin distribution can be obtained [35]. NMR is based upon properties that exist in all molecules, making this detection technique non-destructive. NMR imaging has been applied in many different domains, examples of which are flow through rocks [36] and water transport through trees [37]. These two examples indicate the wide applicability of NMR. Although various articles state that NMR is an in situ and non-destructive biofilm detection technique [34,35], this claim is relative, since the examined biofilm sample must be fixed in a generated magnetic field to perform NMR imaging [34,35,38]. When examining biofilm in bioreactors, the need for a sample to be placed in an NMR spectrometer makes this biofilm detection technique ex situ, and if the technique is applied for bioreactors, it is possibly destructive due to the necessity of transferring the biofilm.

#### 3.1.5. Ultrasonic Time-Domain Reflectometry (UTDR)

Ultrasonic measurements are based on the propagation of sound waves whereby the sound wave velocity (c) through a medium is a function of the mass density and the impedance of the medium [39,40]. At an interface between two media (i.e., the biofilm layer and the surface on which biofouling occurs), the amplitude of the reflected wave depends on the acoustic impedance difference between the media on either side of the interface and the topography of the interface. The impedance, interface properties, and path length may change with the growth of a biofilm layer [39]. This causes a change in the amplitude and arrival times of the sound waves; these changes can be analyzed to quantitatively and in real-time monitor biofouling [39]. Many scientific articles have dedicated their research to the application and biofilm detection properties using ultrasonic time-domain reflectometry (UTDR) [39,40,41,42,43,44]. Li et al. (2006) solely applied UTDR in flat sheet or spiral wound membrane separations. They detected different acoustic response signals from various curved surfaces, and thus successfully detected biofouling in a tubular membrane module. The technique proposed and investigated by Li et al. (2006) [39] was applied for the detection of oil fouling in a hollow fiber membrane [45] and the monitoring of biofilm formation in a wastewater tube [44]. In both articles, the in situ, real-time, and non-destructive detection of biofilm were successfully conducted. Furthermore, the results of the experiments in both articles were 2D- and 3D-visualizations of the biofilm thickness and surface distribution [44,45]. Despite the lack of scientific papers relating UTDR with biofilm detection in bioreactors, Xu et al. (2009) and Wang et al. (2018) provide information that can be used to validate the possible application of UTDR for biofilm detection in bioreactors

#### 3.1.6. Dry Mass Weighing (DMW)

Dry mass weighing (DMW), referred to as the mass per unit area or biofilm density, is used for rapid biofilm growth quantification [46]. To determine the dry mass, the biofilm and its growth substrate (common growth substrate is a glass slide) are placed in an oven at a constant temperature until the water is removed and a constant weight is achieved, hereafter the sample containing the biofilm is cleaned, dried, and weighed again [46,47,48]. This technique is easy to perform and available in all microbiological labs, however, it also has downsides as referred to by Wilson et al. (2017); it does not differentiate between different components of the biofilm such as the EPS and possibly different types of microorganisms, and its usage depends on the heat resistance of the growth substrate [46]. Other researchers have also followed the same procedure with the same consequences, a destructive but in situ detection of biofilm [47,48]. However, the claim of being in situ is arguable concerning the application of DMW for bioreactors. Koo et al. (2003) used a slide as a growth substrate for the cultivation of biofilm [47] and Trulear and Characklis (1982) used an annular reactor [48] containing a removable slide. The technique is applicable for bioreactors; however, the drying step might result in the unbinding of the biofilm of the reactor surface. If this phenomenon occurs, the weight measurements do not differ since all biofilm remains in the bioreactor, however, the critical spots concerning microbial adhesion might disappear. The result of applying DMW will be in mass per surface area or biofilm density [46]. 

#### 3.1.7. Laser-Induced Fluorescence (LIF) Spectroscopy

Laser-induced fluorescence (LIF)-spectroscopy allows for the detection of features not visible to the naked eye or characterization of different substances by utilizing their fluorescence spectral signatures [49]. Fluorescence is the spontaneous emission of radiation by which an atom or molecule relaxes from an upper energy level to a ground state level. The molecules at the surface absorb the photons and become excited. The fluorescence light is emitted when the molecules spontaneously de-excite. The intensity of the emitted fluorescence light can be substantially higher from clean surfaces compared to surfaces that contain biofilm [50]. Furthermore, microorganisms emit light waves with different amplitudes; by using the characterization of these amplitudes, one can differentiate between different species of microorganisms [51]. LIF-spectroscopy is applied in many different domains, an example of the application of LIF-spectroscopy is the detection of fungal growth on high-voltage outdoor composite insulators. In the research of Bengtsson et al. (2005) and Wallstrom et al. (2005), respectively, the aim was to identify critical spots of fungal and biological growth on high-voltage outdoor insulators to eventually overcome the failure of these cables [49,50]. Another example of the application of LIF-spectroscopy is the determination of biofilm contamination of stone structures and cultural heritage [52,53]. In both applications, a laser and a spectrometer are used to obtain information regarding the microorganisms present in the different samples. Despite the lack of information regarding the application of LIF-spectroscopy for the detection of biofilm in bioreactors, LIF-spectroscopy seems to be a useful tool to distinguish different types of microorganisms in bioreactors. The setup of LIF-spectroscopy requires a laser and a telescope connected with a spectrometer, placed on a distance of 1 m and 40 cm, respectively [51]. This setup can also be applied to bioreactors. Moreover, the research of Vieira et al. (2011) utilized a laser diameter of 1.5 cm [51]. To obtain a scan of the complete inner surface of a bioreactor is a time-consuming process. The properties of LIF-spectroscopy make the technique non-destructive and in situ when applied on stone structures and cultural heritage [53] due to the setup of the technique and its application for biofilm detection in bioreactors is also deemed to also be non-destructive and in situ.

#### 3.1.8. Surface-Enhanced Raman Spectroscopy (SERS)

Surface-enhanced Raman spectroscopy (SERS) is a non-invasive analytical tool that combines the molecular fingerprint information provided by Raman scattering with the electromagnetic enhancement power of plasmonic nanoparticles (NPs) [54]. The strong electromagnetic enhancement, typically provided by silver or gold NPs, can provide SERS with extremely high detection sensitivity, down to single-bacteria and even single molecular levels [40]. The claim of Cui et al. (2011) [40], that single molecular detection can be obtained using SERS, is supported by the later research of Chen et al. (2015) [55]. However, to obtain such a high sensitivity, as earlier stated, silver or gold NPs must be used. Research of Kogler et al. (2016) found that cheaper, silver NPs provide a stronger SERS-signal enhancement, however, gold NPs are more stable [56]. The downside of this highly sensitive technique is the cost associated with the NPs. Furthermore, Cui et al. (2011) [40] used SERS to investigate protein fouling on membranes and Kogler et al. (2016) [56] used SERS to investigate biofouling in a flow cell. Both types of research used the same setup; this leads to the assumption that an equal setup can be used when investigating biofouling in bioreactors. However, in both studies, liquid containing biofilm was transferred from its original culturing medium to the growth substrate containing silver and/or gold NPs on the surface. To apply this technique in situ for a bioreactor, the inner surface of the bioreactor must be covered with silver or gold NPs. Moreover, SERS focuses on a small area, thus scanning the entire inner surface of a bioreactor is a time-consuming process. Despite the time-consuming and expensive properties of applying SERS, the technique is capable of in situ, non-destructive, online, and real-time detection without external labeling and on the singular molecular level, thus obtaining information of different species of microorganisms and chemical substances in the same agar medium [57]. 

### 3.2. Chemical

#### 3.2.1. Microtiter Plate Dye Staining (MPDS)

According to Ramajani et al. (2019), microtiter plate dye staining (MPDS) is the most commonly used static biofilm quantification method, which primarily relies on colorimetric dyes (most commonly used are crystal violet (CV) and safranin) that are extracted from stained biofilms [58]. In MPDS, microtiter dishes (most common: 96 well-plate) are cultivated with a bacterial suspension, hereafter, the plates are covered and incubated for a specific time, depending on the experiment [59]. After the incubation time, the plates are washed to remove non-adherent microorganisms. The remaining microorganisms are fixed on the surface, typically by adding a methanol solution [60]. After the addition of the methanol solution, the plates containing the microorganisms are left to dry. If the plates are dry, they are stained. In MPDS, the biofilm can be stained to assess the metabolic activity or to obtain the total biomass. 

#### 3.2.2. Biomass Metabolic Activity

The metabolic activity of a sample containing biofilm is measured to discriminate between living and dead cells. Two stains used to assess the viability of a sample are the Cyanoditolyl tetrazolium chloride salt (CTC) and tetrazolium sodium salt (XTT) [9,61]. To assess the viability of a sample using XTT, a predetermined amount of a reagent solution containing XTT is added, and the metabolic activity of biomass is then measured by the reduction of XTT [9,62]. The measurement of biomass metabolic activity is mostly applied for the quantification of viable cells in planktonic cultures [63]. Another example of a stain used to assess the metabolic activity, however, not in planktonic cultures, is fluorescein diacetate (FDA), in order to measure the total microbial activity in soil and litter [61]. MPDS uses culturing plates to investigate the process of biofilm formation and the activity of the biofilm formed. Thus, it can be applied to investigate what biofilm can form in a bioreactor, that is, when a sample of the liquid of the bioreactor is transferred to a microtiter dish. However, the application to detect biofilm inside a bioreactor cannot be conducted in situ. 

#### 3.2.3. Total Biomass

Like measuring the metabolic activity of the biofilm, the total biomass of a biofilm can be obtained by staining. Two examples of obtaining total biomass by staining are given in Ojima et al. (2016) [59] and Nguyen et al. (2012) [14] where they used a safranin solution to stain *E. coli* cells. After 20 min of rest at room temperature, the microtiter plates were washed twice. After the washing step, the stained cells were solubilized by adding acetone in ethanol, the suspension was condensed, and the index of the biofilm and the number of cells was measured by measuring the absorbance of the dye solution with a microtiter plate reader [14,59]. Another possibility to measure the total biomass produced in a microtiter plate is to apply crystal violet (CV) staining [62]. Marcos-Zambrano et al. (2014) followed the same procedure as Ojima et al. (2016) and Nguyen et al. (2012), but used a spectrophotometer to compute the final measurements of total biofilm. When comparing the different methods, the results vary, thus it can be concluded that MPDS for total biomass has low reproducibility. Furthermore, the colorimetric readouts are most often marred by relatively low sensitivities [58].

#### 3.2.4. Phospholipid Based Biomass Analysis (PBBA)

Phospholipid based biomass analysis (PBBA) is based on the measurement of phospholipids, which are cellular components, as these are universally distributed and expressed at a relatively constant level among the microbial community. However, phospholipid determination is limited by their recovery rate and the sensitivity of the analytical equipment [9]. To identify the different phospholipids, a gas chromatograph is used. This technique is mostly applied in differentiating microorganisms in the soil and providing information concerning the microorganism’s viability and structure [64,65]. However, Azeredo et al. (2017) described this technique as a possible replacement for colony forming units (CFU) [9]. Furthermore, a recent article by Huang et al. (2019) was the first to link microbial respiratory activity with the phospholipid fatty acid of biofilms in full-scale bioreactors [66]. Microorganisms form diverse phospholipid fatty acids (PFA) through various chemical reactions. These chemical reactions vary per species, therefore, PFA is species-specific and can be used to gather information concerning the different species of microorganisms living within biofilms in bioreactors [66]. Furthermore, as stated, it is possible to assess the viability of the microorganisms present in the biofilm. PFA and the oxygen uptake rate (OUR) are both discovered solely in living cells and can thus be used as characteristic biomarkers for living microorganisms [66]. The downside of the application of Huang et al. (2019) is that the bioreactor in question was a bioreactor used for wastewater treatment plants. The goal of their specific research was to obtain information regarding the number of living microorganisms present in the water and eventually irradiate most of them. In contradiction, in a bioreactor used to produce algae, the solution present in the bioreactor consists mostly of microorganisms and the goal is to obtain more algae. From this, it can be concluded that phospholipid-based biomass analysis can be used to differentiate between microorganisms present in a specific medium. Furthermore, it can estimate the viability of the cells present in a medium. However, the application of phospholipid-based biomass analysis for the estimation of cell viability is dependent on the process that the bioreactor in question intends to execute. 

### 3.3. Microscopical

This section elaborates on the different microscopy techniques and their similarities and differences. An important characteristic of a microscopy technique is its ability to enlarge a specific sample. For this specific characteristic, the techniques were compared using their respective magnification. 

#### 3.3.1. Light Microscopy

Light microscopy is a useful base-line technique to provide visual identification of biofilm formation [9]. Light absorption by biofilms was found to correlate with biofilm cell mass and total biofilm mass. Light microscopy is based on the linear relation between the intensity of a pixel in biofilm images and the corresponding number of cells. This relation allows the calculation of biofilm thickness [67]. Light microscopy requires simple sample preparation and is cheap and easy to perform. However, compared to other microscopy techniques, its resolution is relatively low. This enables the imaging of larger parts of a sample compared to other microscopy techniques, however, it also has its downsides: its resolution is not high enough to determine inter-cellular relationships and morphotype differentiation [9]. The application of light microscopy results in an image of the biofilm. Furthermore, the visualization of biofilm by light microscopy requires staining of the biofilm. Previous research by Harrison-Balestra et al. (2003) used Congo red staining as a means to visualize the formation of biofilm in wound tissue [68]. According to Azeredo et al. (2017), the cheapest and most effective recently developed staining methods are Hematoxylin and Eosin, periodic acid–Schiff, and Brown and Brenn Gram staining [9]. Thus, to successfully apply light microscopy, first a suitable staining method must be selected. 

#### 3.3.2. Confocal Laser Scanning Microscopy (CLSM)

Confocal laser scanning microscopy (CLSM) is fluorescence microscopy (i.e., it uses staining to obtain several parameters of biofilm). To visualize components of EPS by CLSM: (1) Carbohydrates can be stained where the patrons of the stains obtained depend on the specificity of the biofilm; (2) Proteins present in the biofilm can be stained; and (3) eDNA can be stained [69]. Moreover, CLSM is the most widely used fluorescence microscopy to study biofilms [70] as it allows for the evaluation of the spatial structure of the biofilm and the visualization of cell distribution on the biofilm matrix. The application of the technique results in 3D images of the biofilm and parameters such as biofilm thickness and biofilm roughness [70]. The 3D images are created by a computer processing a series of XY and XZ plane optical sections [71]. Unlike many other microscopy techniques, CLSM does not require fixation and dehydration of the biofilm sample, thus it is a non-destructive technique that can be performed in situ and in real-time [71]. However, the claim of being in situ is only valid for biofilm samples formed on a flat surface that will fit under the microscope. Thus, applying CLSM to detect biofilm in bioreactors is deemed to be ex situ. Moreover, CLSM has been applied in many different domains, examples of which are the detection of biofilm on dairy industrial reverse osmosis membranes [72], in validating anti-fouling properties of specific polymers [73], and the identification of marine bacteria and their biofouling characteristics [74]. The images obtained by applying CLSM in the different domains are of alternating resolution; the highest resolution (scale bar) obtained by Stoica et al. (2018) is 101 μm, Boguslavsky et al. (2018) acquired a resolution of 50 μm (scale bar), and Jeong et al. (2018) were able to obtain a maximum resolution of 20 μm (scale bar). When comparing these results, it can be concluded that the difference in resolution is caused by the usage of microscopes constructed by various manufacturers. 

#### 3.3.3. Scanning Electron Microscopy (SEM)

Scanning electron microscopy (SEM) is a microscopy technique based on surface scattering and the absorption of electrons achieving high depth, yielding a 3D appearance of the biofilm surface, visualization of the biofilm, distribution of the biofilm, and EPS dispersed on the biofilms [75,76]. To visualize these characteristics, drying the prepared sample and operating an ultrahigh vacuum is necessary [44,75,77]. Several examples of the application of SEM are to study the ability of bacteria to develop biofilms on different surfaces in several environmental conditions [78], research the temperature and surface material dependence of Salmonella spp. biofilm [79], and the performance of grafted nanosilica as an anti-biofouling polymer [73]. These three different examples indicate the wide applicability of SEM. Due to the properties of SEM to enlarge samples as much as up to a single molecular level, the adhesion properties of single microorganisms can be monitored [73]. This property makes SEM an ideal technique to investigate possible biofouling repellent materials. According to Norton et al. (1998), SEM is capable of visualizing a very thin biofilm because it focuses on the surfaces of objects. This is favorable when early biofilm formation is investigated, however, when the biofilm formation is in a later growth stage such as the proliferation of the microorganisms, the images obtained by SEM remain focused on the top layer of the biofilm and thus do not provide any information with respect to the thickness of the biofilm or its 3D structure [80]. However, later research of Clayborn et al. (2015) found that the application of SEM is capable of providing a 3D visualization of the biofilm [75]. When comparing both articles, different microscopes produced by Phillips were used. However, this does not explain the difference in detection properties. Clayborn et al. (2015) made use of an image processing technique that generated a 3D reconstruction of the biofilm. It can thus be concluded that SEM by itself is not capable of generating 3D images of biofilms. Although SEM is not capable of visualizing the thickness and the 3D structure of the biofilm, it can provide a spatial resolution of up to 10 nm [81]. However, Doucet et al. (2005) did not focus on the application of SEM for biofilms [81]. Moreover, Chatterjee et al. (2014) and Merino et al. (2019) stated that the maximum resolution that can be obtained by the application of SEM to image biofilms was 50 nm [77,78]. Since SEM uses the wavelength of electrons to image the stated biofilm properties, obtaining a resolution higher than 50 nm is not possible. Any attempt in obtaining better resolution results in energies that immediately damage biofilm samples [77]. Despite the high resolution, SEM is not capable of differentiating between different microorganisms, therefore the microorganism of which the biofilm consists must be known beforehand. For any application of SEM, extensive sample preparation is necessary. The required sample preparation might result in damaging the soft biological samples and can cause artifacts [77]. Thus, if SEM is applied for bioreactors, a biofilm sample must be removed from the bioreactor and prepared, which might result in damaging the sample, thus SEM is a destructive and ex situ biofilm technique. 

#### 3.3.4. Atomic Force Microscopy (AFM)

Atomic force microscopy (AFM) is a microscopy technique based on the deflection of a metallic “tip”. This metallic tip moves over the target surface, and the deflection of the tip is recorded [82]. Utilizing the recorded deflection, the topology and material properties of a surface can be measured. AFM is a non-destructive technique and is capable of obtaining 3D topographic views, biofilm structural details, and various interactions such as microorganism–surface interaction forces and biofilm cohesion [77,78]. Except for these properties, Phang et al. (2009) applied AFM to study the nanomechanical properties such as strength, elasticity, and toughness of biomacromolecules at the single-chain level [83]. Moreover, in contrast to other microscopy techniques, AFM can be applied under ambient conditions and therefore renders pre-treatment of samples obsolete [77]. AFM is also applicable on liquid surfaces, which is generally necessary in the in situ imaging of biofilms [84]. However, to apply AFM on liquid samples, the procedure must be changed. If the tip moves over the surface, the tip might damage the biofilm. To overcome the biofilm being damaged, the constant movement of the tip across the biofilm surface is changed to intermittently tap the surface [77]. The technique of tapping, instead of constantly moving across the biofilm surface, is widely used [85,86]. Despite the claim of Merino et al. (2019) that AFM is a non-destructive biofilm detection technique, Birarda et al. (2019) stated that to evaluate the matrix thickness, part of the matrix was scratched and the thickness difference between the scratched area and the biofilm area was measured [87]. The application of AFM by Birarda et al. (2019) indicates that the technique is destructive if the aim of applying AFM is to obtain information concerning the biofilm thickness of a sample. Furthermore, compared to other microscopy techniques, AFM is capable of offering the highest resolution of 1–10 nm [78], and according to Chatterjee et al. (2014), AFM can provide nanometer resolution almost routinely [77]. Although AFM is not limited by extensive sample preparation, when it is applied to detect a biofilm formed in a bioreactor, the biofilm must be transferred to a sample on which the microscope can focus. Therefore, like all other microscopy techniques, the application of AFM for bioreactors is ex situ and might also be destructive. 

#### 3.3.5. Transmission Electron Microscopy (TEM)

Transmission electron microscopy (TEM) observations are conducted by measuring the elastic and inelastic interactions of an electron beam that is transmitted through a specimen [88]. The electron beam is housed in a vacuum environment to minimize unwanted electron–gas interactions. Therefore, the perfect sealing of the liquid biofilm cells is a prerequisite for successful imaging. Additionally, the sample that is used in TEM should be thin enough to minimize electron-beam scattering and ensure the high resolution of TEM observation, with the thickness of the TEM samples lying generally below 150 nm [76,88]. Through the use of TEM, the internal cross-sectional detail of the individual microorganisms and their relationship to each other including the overall biofilm can be visualized [89,90]. However, TEM focuses on a very small area, thus the claim of being capable of visualizing the overall biofilm is only relative. Like SEM, TEM uses electron beams to visualize objects. As stated, SEM is not capable of providing a resolution higher than 50 nm, since the required wavelength of electrons to achieve this results in damaging the biofilm sample [78]. However, according to Lawrence et al. (2003), TEM is capable of providing a practical resolution of up to 1 nm if the sample is prepared according to nanoplast preparations, and 3 nm if the sample is prepared according to epoxy preparations [90]. The cause of these alternating practical resolutions is that TEM uses transmitted electrons, the electrons that pass through the sample before they are collected [76]. Moreover, unlike SEM, sample preparation to apply TEM is a time-consuming process and a tedious procedure that requires trained laboratory workers, which is due to the necessity of a very thin sample, a vacuum environment, and the absence of artifacts such as precipitates or amorphization [76,89]. Generally, TEM can be used on hydrated biofilms; however, in practice, multiple articles state that drying is necessary to obtain a sample thickness with a maximum of 150 nm [76,81,91]. Since a biofilm in a bioreactor is hydrated, this technique is deemed to be ex situ for bioreactor applications. Although it is a microscopy technique, TEM requires the drying of a sample, thus it also a destructive technique if applied to bioreactors. 

#### 3.3.6. Environmental Scanning Electron Microscopy (ESEM)

According to Ramajani et al. (2019), MPDS is the most commonly used static biofilm quantification method, which primarily relies on colorimetric dyes (most commonly used are crystal violet (CV) and safranin) that are extracted from stained biofilms [58]. 

According to Surman et al. (1996), environmental scanning electron microscopy (ESEM) is a modified form of SEM, however, recent literature has stated that ESEM is a separate instrument and in most cases is not a modification of SEM [92]. The high water pressure used in ESEM enables imaging of the hydrated specimen, unlike SEM, which can only image dry samples [14,89]. Furthermore, it does not depend on the high vacuum requirements like SEM [81]. The direct study of fully hydrated or electrically non-conductive dry samples in their native state, without the necessity of a conductive coating, is possible due to high gas pressure, mostly water vapor, in the ESEM specimen chamber [92]. The most important benefit of ESEM, in comparison with its predecessor SEM, is the capability of dynamic in situ investigation of sample changes or reactions under various temperatures and pressures [93]. According to Doucet et al. (2005), ESEM can provide a resolution of up to 30 nm [81]. However, Doucet et al. (2005) also claim that SEM can provide a resolution of 1 nm. The research of Doucet et al. (2005) focused on the visualization of natural aquatic colloids and particles, and the different focus could be used to explain the difference in maximum resolution obtained by Chatterjee et al. (2014) and Merino et al. (2019) [77,78] and the respective maximum resolution obtained by Doucet et al. (2005) [81]. However, to obtain a maximum resolution of ESEM concerning the visualization of hydrated biofilm characteristics, articles that apply ESEM for biofilm visualization were consulted. Callow et al. (2003) imaged the spore adhesive of marine algae in its natural state [94], however, this article does not provide insight concerning the maximum resolution of ESEM for biofilm imaging. Another article that used ESEM to visualize and qualify between different species of microbial biofilms was that by Priester et al. (2007) [95]. Priester et al. (2007) used ESEM to visualize native morphologies including surface structures. Since ESEM minimizes biofilm dehydration, it preserves the stated native structures. However, like Callow et al. (2003), Priester et al. (2007) did not supply any information as to the maximum resolution of ESEM. Thus, due to the lack of information, it is assumed that for the visualization of biofilms, the maximum resolution of ESEM will be larger than 50 nm. Furthermore, the sample preparation for ESEM is rather fast compared to most other microscopy techniques. ESEM does not require staining, drying, or coating of samples. This makes ESEM beneficial with regard to time consumption of the biofilm visualization process and causes significantly less disruption and damage to the biofilm sample [81]. Like other microscopy techniques, the application of ESEM for bioreactors requires sample preparation. The sample preparation requires the biofilm formed in the bioreactor to be removed, which might cause damage to the biofilm; however, due to the absence of necessary drying and staining of the sample, no damage is done later on.

#### 3.3.7. Scanning Transmission X-Ray Microscopy (STXM)

Scanning transmission x-ray microscopy (STXM) is a powerful tool, in which chemical sensitivity is achieved through the near edge x-ray absorption spectrum (NEXAFS), and utilizing these NEXAFS, it can be applied to fully hydrated samples [90,96]. This is possible due to the ability of soft x-rays to penetrate water, the presence of suitable analytical core edges in the soft x-ray region, and reduced radiation damage compared to electron beam microscopy techniques [90]. STXM uses the intrinsic x-ray absorption properties of the sample, thus eliminating the need for the addition of probes and/or markers that might damage or complicate the sample. STXM makes a collection of a sequence of images, which over a range of energies, supply detailed mapping of chemical species [90]. However, to successfully apply STXM, a list of bonding structures of chemical species beforehand is necessary [97]. Since STXM is an x-ray absorption technique capable of providing both chemical and biochemical information, the early application of STXM mostly focused on mapping the chemical information of microbial biofilms such as the different species of iron and metal present in these biofilms [96,98]. However, STXM has also been applied for visualizing other aspects of biofilm such as the mapping of the EPS matrix of microbial biofilms [90], the early stages of biofilm formation [99], and the imaging of micro-processes in biofilm matrices [100]. Furthermore, STXM uses the intrinsic x-ray absorption properties of the biofilm sample, therefore there is no need for adding any reflective, absorptive, or fluorescent probes that might cause damage or artifacts to the biofilm sample [97]. Despite these non-destructive characteristics of STXM, it also has some characteristics that might be destructive for biofilm samples by damaging or causing adverse effects by radiation and x-ray absorption saturation [97,100]. Another great advantage of STXM is that unlike SEM, it uses the electrons that are repelled by the molecules within the biofilm. This leads to the capability of obtaining a maximum spatial resolution of 25 nm [101]. For STXM, its maximum sample thickness is 30 μm [97], which is significantly larger than most other microscopy techniques. Despite this large allowance for sample thickness, this technique is still not capable of performing in situ imaging of the biofilms formed within a bioreactor. Thus, the removal of the biofilm is necessary, which might damage the biofilm sample. 

### 3.4. Biological

A biofilm detection/measuring technique is referred to as biological if the technique targets a specific biological characteristic of the microorganisms of which the biofilm consists.

#### 3.4.1. Determination of Colony-Forming Units (CFU)

Colony-forming units (CFU) is the most widely used technique to estimate biofilm cell viability [9]. The basic concept of this assay is to separate the individual cells on an agar plate and grow colonies from cells, therefore differentiating living from dead cells. CFUs are a measurement of how many predecessors are present in a given population of cells; if an individual cell can proliferate and divide into mature cells, it will make an individual colony [46]. However, according to Li et al. (2014), CFU comes with some risks. Viable but non-culturable (VBNC) cells are characterized by a loss of culturability on routine agar, which impairs their detection by CFU [12]. According to Li et al. (2014), it is even possible that all bacteria in a sample are in the VBNC state. If this phenomenon occurs, the sample may be regarded as germ-free due to non-detection [12]. The procedure starts with a mature biofilm that is transferred to a liquid medium via scraping, vortexing, or sonicating and is thus a destructive, ex situ biofilm measuring technique if applied for bioreactors. After incubation in the liquid medium, colonies are counted on the plates and the number of cells per mL (CFU/mL) of the original culture is calculated using mean colony counts, the volume of culture plated, and the dilution factor from the suspended biofilm [46]. 

Except for measuring viability using the number of CFU, this technique can also be used for other purposes. An example of this is the application to test whether different materials affect the growth of the microorganism, that is, when transferred from a medium to culture plates of two or more different materials and compared to those [102]. Akens et al. (2018) [102] applied CFU to compare stainless steel and titanium orthopedic plates. Stainless steel is one of many construction materials for bioreactors and therefore it is assumed that the technique can also be applied to assess several possible construction materials for bioreactors. Another application of CFU is to assess the performance of several anti-biofouling materials [103]. The result obtained by performing the determination of CFU for cell viability will be in the form of a graph containing the number of cells per mL. The CFU technique typically does not require highly specialized or advanced equipment, therefore it can be performed in every microbiological lab [9,46]. The technique also has its downsides; it is time and labor-intensive and there exists a large possibility for errors to occur due to scraping and counting [9,46]. 

#### 3.4.2. Light Microscopy

QPCR (quantitative polymerase chain reaction) allows for the measurement of microorganisms efficiently and rapidly with specific and sensitive detection. It is designed to quantify microorganisms by directly targeting genomic DNA and can yield results within a few hours by eliminating steps requiring time-consuming incubation [104]. Moreover, qPCR is a technique that is widely applied; examples of the application of qPCR are the detection of bacterial biofilm in breast implants [105], the analysis of multi-species oral biofilms (Suzuki, et al., 2005) [106], and the detection of Enterobacter cloacae strain in a bioreactor for chromate wastewater treatment [107]. In addition to the detection of the Enterobacter cloacae strain, Nozawa et al. (1998) also used qPCR as a quantification method for other specific microbes present in the wastewater. According to Klein et al. (2012), a drawback of qPCR is its tendency toward overestimating the number of viable cells due to the presence of DNA derived from dead cells and free extracellular DNA (eDNA) [108]. To solve this problem, treatment with propidium monoazide (PMA) has been proposed. PMA only enters membrane-comprised cells. During PMA-qPCR, cells that have been affected by PMA will not be amplified [9]. However, PMA-qPCR has some drawbacks: the discrimination between viable cells and dead cells is based on membrane integrity, thus the presence of antimicrobials that do not affect membrane integrity cannot be monitored [9]; slightly damaged cells that are still viable may not be accounted for; and the presence of PMA-binding compounds in the sample can prevent efficient PMA-DNA binding [9]. Compatible to Klein et al. (2012), Suzuki et al. (2005) encountered a problem with the quantification of the oral biofilms caused by contamination, interfering substances, and unequal amounts of collected samples [106]. Instead of applying PMA-qPCR, Suzuki et al. (2005) used a TaqMan probe, which is a fluorescent DNA probe, to overcome these problems. Using this TaqMan probe, Suzuki et al. (2005) were able to provide rapid, sensitive, and quantitative detection of multiple species of microorganisms. Furthermore, the usage of qPCR in combination with this TaqMan probe resulted in real-time and in situ monitoring of biofilm activity and microorganism diversity. However, qPCR also has some drawbacks. TaqMan real-time qPCR requires a list of biofilms beforehand. Another drawback might be the application of TaqMan real-time qPCR for bioreactors; due to a lack of literature, it cannot be stated whether TaqMan real-time qPCR is applicable for bioreactors. Suzuki et al. (2005) applied the method for the analysis of oral biofilms. Further research should provide information on the applicability of TaqMan real-time qPCR for bioreactors. 

### 3.5. Combinations of Different Categories

#### 3.5.1. Extracellular Polymeric Substance (EPS) Extraction

Ex situ EPS extraction protocols are based on physical methods (e.g., steaming, heating, high-speed centrifugation, ultrasound) and/or chemical reagents (e.g., ethanol, NaOH, formaldehyde, pH adjustments, ethylenediaminetetraacetic acid) [9,109]. The selection of one of these methods depends on the biofilm species and the complexity of EPS. However, in general, the application of solely chemical methods increases EPS yields compared to the application of solely physical methods [9]. As stated, there does not exist a universal EPS extraction method for all different types of biofilm. Therefore, many studies have focused on the EPS extraction of one specific biofilm, examples of which are the research of Yang et al. (2019) the focused on EPS extraction of Geobacter biofilms and Wu et al. (2019), who investigated artificial soil biofilm formation and used a chemical reagent, cation exchange resin to extract the EPS [110]. EPS extraction is labeled as a combination of different categories due to its need for a microscope to, once extracted, examine the EPS. To apply EPS extraction for biofilms formed in bioreactors, first a proper extraction protocol must be selected, which depends on the biofilm species and the complexity. However, according to Azeredo et al. (2017), there is another important factor before selecting a specific protocol, the scientific question to be addressed. If one aims to investigate ion binding characteristics, extraction by chemical reagents should not be selected, since this might influence the binding of its strength. EPS extraction is, for both bioreactor and other applications, ex situ and destructive, therefore it should only be selected when the focus is on specific aspects of the EPS. 

#### 3.5.2. Anti-EPS Component Antibodies

Antibodies can be used to detect some specific fibrous strands of EPS in biofilms [9]. To successfully apply this approach, the specific proteins that constitute the biofilm matrix must be identified beforehand. Once the proteins are identified, antibodies that specifically target these proteins must be selected and produced [9]. The production costs of these antibodies are high, however, this approach could be valuable to locate and, combined with a microscopy method, image-specific components in the biofilm EPS matrix. Apart from locating specific components of the biofilm, Ryser et al. (2019) found that a specific antibody that exists in the human body extracts key scaffolding proteins from the biofilm matrix [111] and thus has properties that counteract biofouling. However, the current literature only relates these specific antibodies with biofilms in the human body and not with biofilms in bioreactors. Another example of the application of anti-EPS component antibodies is provided in the research of Carrano et al. (2019) [112]. Carrano et al. (2019) used germ tube antibodies to reduce the growth and biofilm formation of *C. Albicans* [112]. In the research of Carrano et al. (2019), the focus was specifically on proving that the specific antibody influenced the growth and biofilm formation. To prove the anti-fouling properties of the antibody, several chemical, physical, and microscopic methods were applied. 

#### 3.5.3. Fourier Transform Infrared (FTIR) Spectroscopy

Fourier transform infrared (FTIR) spectroscopy is a widely used method due to its robustness and sensitivity. It uses infrared radiation, which is a non-invasive and non-destructive type of radiation [113]. IR causes vibration of the covalent bonds of components of the biofilm. The different components of the biofilm vibrate differently at characteristic frequencies, resulting in a unique spectrum for each sample [113]. An example of the application of FTIR spectroscopy is the continuous non-destructive monitoring of biofilms in continuous flow chambers [114]. According to Serra et al. (2007), FTIR spectroscopy is capable of following the dynamics of biofilm growth in continuous/in real-time. This claim is relative since the specific research removes plates from the flow chamber every 24 h. After removal, the plate is washed, dried, and re-suspended in sterile distilled water. Hereafter, the carbohydrate-to-protein ratio is obtained. In this case, the increase in the carbohydrate-to-protein ratio is a marker for biofilm growth. Another example of the application of FTIR spectroscopy is to evaluate patient evolution regarding chronic wounds [115]. Exudate from a chronic wound is removed and placed under a spectrometer, with the goal to identify different proteins and bacteria and draw conclusions regarding the time-varying quantities of the proteins and bacteria. The exudate from the wound is mixed with water and a comparable procedure to Serra et al. (2007) is followed. However, Ceruscio et al. (2018) did not claim to continuously/in real-time monitor the stated chronic wounds. However, another example is the study of Singhalage et al. (2018), which is the first article to apply FTIR spectroscopy to study the modifications on the cellular structure of fungal biofilms [116]. The fungal biofilms were formed in a biofilm-forming medium. At specific time intervals, samples were taken and analyzed using an FTIR spectrometer. This research neither claims to be continuous or in real-time. Moreover, from the given examples, it can be concluded that FTIR spectroscopy allows one to monitor the complete molecular diversity (i.e., lipids, proteins, carbohydrates, and nucleic acids) on the same surface [14,113]. Another advantage of FTIR spectroscopy is that it is a quick, easy to use, and inexpensive method compared to many other techniques [116]. Despite the good properties, FTIR spectroscopy is only capable of providing information on the base layer (surface) of biofilms [115] and is thus not capable of providing information concerning the 3D-structure or biofilm thickness. The results obtained by applying FTIR spectroscopy will be in the form of a graph comparing the different components of the biofilm using their alternating characteristic frequencies.

### 3.6. Combinations of Different Categories

This section combines the literature found for all different detection techniques into a table that can be applied to compare detection techniques. Table 1 can be used by scientists to study their biofilms. 

### 3.7. Properties of Detection Techniques

This section summarizes the different properties and results produced by the various detection techniques. Table 2 serves as a tool for researchers so that they can easily select a biofilm detection/measuring technique to study their biofilms. 

## 4. Confluence of Material in Biofilm Formation

### 4.1. Construction Materials for Bioreactors and Their Effect on Biofouling

A bioreactor is a vessel that provides an environment suitable for the controlled growth of a pure culture or a defined mixture of organisms [132,133]. The construction material of a bioreactor may not adversely affect, nor be adversely affected by, the desired microbial activity. Furthermore, the construction materials for bioreactors must be resistant to corrosion by the nutrient medium and products and to the effects of sterilization temperatures, and the construction material must be able to withstand the stresses produced by the pressure in the bioreactor [133,134]. 

Moreover, microorganisms can colonize virtually every environment [19], thus biofouling may occur on every single component of the bioreactor. However, evaluating all these components is a time-consuming process. Therefore, this study focused on biofouling on the inner surface of the reactor vessel and the agitator, so only the construction material of the inner surface of the reactor vessel and the agitator were taken into consideration. Bacterial adhesion is controlled by the hydrophobicity as well as the negative electrokinetic potential of the cell [14,134]. Aside from these properties of microorganisms, some properties of the material itself also influence biofouling. To provide a literature background, Section 4.2, Section 4.3, and Section 4.4 focus on the material properties of the conventional and respectively state-of-the-art construction materials for bioreactors. An important aspect of the material used to construct a bioreactor is whether the material contributes to or counteracts the process of biofouling. Nyugen et al. (2012) [14] have dedicated their research toward biofouling on water treatment membranes and provided a table with all of the factors affecting microbial adhesion to membrane surfaces including factors caused by microorganisms and the feed water of the reactor. However, these factors might differ for membrane surfaces and reactor vessel surfaces, therefore supportive literature research was undertaken. Achinas et al. (2019) considered surface charge, hydrophobicity, surface roughness, and surface topographical configuration as specific properties of materials that can affect biofouling [7]. Nguyen et al. (2012) also included conditioning film, surface tension, chemical composition, and porosity, however, these are properties related to membranes, and thus not included in Table 3.

Moreover, Vanysacker et al. (2014) also studied the differences between membrane fouling and biofouling in natural ecosystems [6]. Their research concluded that the initial attachment was mostly dependent on the transmembrane pressure (TMP) for biofouling on membranes and mass transport, thermal, and gravity effects for biofouling in natural ecosystems. This research focused on biofouling in bioreactors, and bioreactors are far from natural ecosystems, however, the authors indicated that factors that affect membrane biofouling are not per se factors that affect biofouling in bioreactors.

### 4.2. Stainless Steel as a Construction Material for Bioreactors

In the introduction, it was stated that conventional construction materials for bioreactors are stainless steel (SS) and glass [4]. To assess whether the different types of conventional construction materials affect microbial adhesion, the construction materials were compared based on the literature providing information for the four entries under the heading “surface” in Table 3. There exist five “types” of stainless steel; ferritic, austenitic, martensitic, duplex, and precipitation hardening. These so-called “types” refer to the microstructure of the steel [135]. However, this research focused on the collection of all stainless-steel types and focused on their overall instead of their individual properties. 

The surface charge is a factor that can affect biofilm formation [5]. According to Landoulsi et al. (2011) [20], the distribution of surface charge is dependent on the pH-value of the medium; an SS surface is negatively charged in natural water with a pH-value of approximately 6–8. The pH-value of the medium depends on the application of the bioreactor. Moreover, they stated that point zero charges (PZC) of SS will be around a pH of 3–4. From these values, it can be concluded that the surface charge of SS becomes more positive with a decreasing pH-value. If the surface charge is negative and the microorganisms are also negatively charged, a repulsive force between the two occurs. According to Achinas et al. (2019), most bacterial cells are negatively charged, therefore, if the bacterial cells are negatively charged, a negative surface charge is preferred so that repulsion occurs between the surface and bacterial cells.

The hydrophobicity of a surface affects the number of attaching microorganisms [7]. According to Parkar et al. (2001) [136], who researched the attachment of thermophilic bacilli to SS surfaces, microorganisms have a greater tendency to attach to hydrophobic rather than hydrophilic surfaces and, like all other metals, stainless steel has a hydrophilic surface. However, Parkar et al. (2001) [136] also concluded that thermophilic bacilli can more readily attach to stainless steel surfaces than vegetative cells. Thus, it can be concluded that the hydrophilic properties of a SS surface do not exclude microbial attachment. Furthermore, (Palmer, et al., 2007) [137] concluded that the hydrophobicity of bacterial cells was dependent on the molecules existing on the cell surfaces. Examples of molecules that affect hydrophobicity are proteins and lipids. 

Akens et al. (2018) compared the growth of *Staphylococcus aureus* biofilm on SS and titanium orthopedic plates [102]. Aside from the comparison of titanium and SS as construction materials, they also applied thermal cycling on SS plates and compared them with plates without thermal cycling. The thermal cycling procedure resulted in lower surface roughness. To compare thermal cycling SS plates and non-thermal cycling SS plates, CFU was applied to obtain the number of bacteria capable of forming colonies. Their results showed that surface roughness affected biofilm formation. Non-treated plates contained 10^10^ CFU and treated plates 10⁸·⁵ CFU. Thus, from this research, it can be concluded that the surface roughness indeed affects biofouling and that the surface roughness of SS can be decreased by treatment. To be able to compare the surface roughness of SS with other materials, a value must be obtained. In this research, we focused on the maximum obtainable surface roughness, which for SS is <0.01 μm [138].

The surface topographical configuration (STC) can be seen as the distribution of the surface roughness over the surface. Perhaps a better explanation is provided by Achinas et al. (2019) as the way that peaks and valleys are distributed along the surface. Jullien et al. (2003) imply that the STC of SS has little influence on the initial attachment of microorganisms, however, it has a larger effect on later stages such as biofilm development [139]. This is caused by the protection of cells from removal, thus providing a more stable environment for biofilm growth [139]. However, according to Li et al. (2018), the STC of SS can be drastically changed by heating, melting, plasma formation, and vaporization [140]. Therefore, no general conclusion can be made regarding the STC properties of SS. 

### 4.3. Glass as a Construction Material for Bioreactors

There exist many different types of glass, however, this research does not focus on one specific type of glass, as our attention was on the glass that has already been applied for the construction of bioreactors (i.e., glass that has already been researched by others).

Moreover, the selection of a material for a bioreactor partly depends on the purpose of the bioreactor. An example is provided by the article of Uyar, B. (2016). In his research, Uyar aimed to select a construction material for a photobioreactor. For a photobioreactor, the transmittance of light is a necessity, therefore glass was selected as a construction material [141]. Moreover, like Uyar, the research by Zeriouh et al. (2017) [142] aimed to design a photobioreactor (PBR) that did not suffer from microbial adhesion since biofouling in a PBR reduces the light transmittance and thus the desired reaction. Within this paper, the glass will be compared based on the same entries as SS, as stated in Table 3 under the heading “surface”. As stated previously, the surface charge of the material that is applied for the construction of the bioreactor can influence the adhesion of microorganisms [5]. Research by Marques et al. (2007) submerged both twenty SS and glass chips in pH neutral liquid containing *Staphylococcus aureus*. The results obtained by the research of Marques et al. (2007) presented a higher intensity of biofilm formation on the glass chips rather than the SS chips. This higher intensity of biofilm formation on glass may be explained by the higher electrical or surface charge of the glass chips [143]. The research of Marques et al. (2007) implies that glass has a higher, thus more positive, surface charge than SS. To substantiate the claim of Marques et al. (2007), additional research in the literature resulted in obtaining the PZC of a borosilicate glass, which is around a pH-value of 3 (Amadu & Miadonye, 2017) [144]. The claim of Amadu and Miadonye contradicts the information stated in the research of Marques et al. (2007) and Landalousi et al. (2011). 

According to Landalousi, SS has a PZC of around pH 3–4 and according to Marques et al. (2007), the surface charge of glass is higher than that of SS whilst Amadu and Miadonye (2017) claimed that glass had a PZC of approximately 3. It is difficult to explain this contradiction since both experiments used different setups and the glass chips used in the research of Marques et al. (2007) are referred to as glass and not explicitly as, for example, borosilicate glass. The second entry in Table 3 is Akens hydrophobicity. According to Zeriouh et al. (2017), glass has hydrophilic properties, however, the same article also claims that SS has hydrophobic properties whilst Parkar et al. (2001) claimed SS to be hydrophilic. Moreover, unlike Parkar et al. (2001), Zeriouh et al. (2017) stated that the tendency of microorganisms to adhere to surfaces did not rely on the hydrophobic or hydrophilic properties of the material, but also on the microorganism in question (e.g., the microalgae species *N. closterium*, unlike most other microalgae has a weak attachment to hydrophobic substrates [142]. A perfectly unblemished glass surface allows water to adhere and is therefore hydrophilic. However, if any form of contamination of the glass occurs, it becomes hydrophobic [141]. 

From the research of Akens et al. (2018), it can be concluded that the surface roughness of the construction material of the bioreactor influences the adherence of microorganisms on the reactor wall. Han et al. (2017) [145] rephrased this as follows: the surface roughness influences the wetting nature of materials and surfaces. The larger the surface roughness, the more likely it is that larger numbers of microorganisms adhere to the surface. Like SS, the surface roughness of glass can also be decreased. One of the techniques to lower the surface roughness of glass is polishing [146], which can succeed in reducing the surface roughness of 344 nm to less than 40 nm [146]. Comparing these numbers with the surface roughness of SS, we can conclude that the surface roughness can be lower than that of SS. The STC of glass is equal to that of SS, depending on the treatment of the surface. Therefore, no claims can be made based on the STC of glass. If glass is used as a construction material for a bioreactor, specific research must be conducted into the STC of the glass in question. 

### 4.4. Resin as An Alternative Construction Material for Bioreactors

3D printing materials can be used for multiple applications, showing advanced properties as stated in the literature [147,148,149,150,151,152,153]. However, there is not enough supportive literature to compare the specific FLGPCL02 proprietary resin with SS and glass. Therefore, the claims are based on literature that contains information concerning any type of resin. 

According to Landoulsi et al. (2011), the surface charge depends on the pH value of the medium [20]. The current literature does not support a claim regarding the surface charge of FLGPCL02 proprietary resin. However, a study by El Khoury et al. (2016) [154] applied electrostatic force spectroscopy (EFS) to obtain the surface charge of several epoxy resin materials common in electrical engineering. El Khoury et al. (2016) concluded that the surface charge of these epoxy resin materials was positive. We proposed using a similar setup as El Khoury et al. (2016) to obtain the surface charge of the FLGPCL02 proprietary resin specifically. The downside of this setup is that it is executed in open air, which should be taken into consideration whilst obtaining the surface charge of FLGPCL02 proprietary resin. 

Composites of resin are materials consisting of a hydrophobic resin matrix and less hydrophobic filler particles [155]. This implies that the surface of a resin composite is hydrophobic, however, the hydrophobicity is not equally distributed across the surface. Parkar et al. (2001) stated that microorganisms have a greater tendency to attach to hydrophobic rather than hydrophilic surfaces. Thus, the hydrophobic properties of resin encourage the adhesion of microorganisms. 

The research of Ono et al. (2007) [156] compared three types of resin on their surface properties concerning *Streptococcus mutans* biofilm formation. The three resin types used within the research of Ono et al. (2007) were Clearfil AP-X, Grandiom, and Reactmer Paste with a surface roughness of 0.25 ± 0.66 µm, 0.22 ± 0.01 μm, and 0.23 ± 0.01 µm, respectively. Comparable to the research of Akens et al. (2018), a larger surface roughness within the resin composites resulted in more bacterial adherence. However, instead of using CFU as a detection technique Ono et al. (2007) compared the amount of biofilm using SEM images. Like SS, it is also possible to decrease the surface roughness of resin, where a possible technique to obtain a lower surface roughness is polishing [155,156]. Moreover, Shimokawa et al. (2019) researched the properties of several different bulk-fill resins as dental prosthetics and concluded that the surface roughness of the different resin composites can easily be increased by a toothbrush [157]. This implies that detection techniques that require scratching the biofilm of the surface of a bioreactor might increase the surface roughness of bioreactors constructed of FLGPCL02 proprietary resin. However, to support this claim, further research must be conducted. 

Equal to SS and glass, the STC can be seen as the distribution of peaks and valleys along the surface. Therefore, the distribution of the stated peaks and valleys is dependent on the accuracy or maximum resolution of the printer. Based on the literature provided in the STC section of SS, the STC of resin can also be drastically changed. 

## 5. Conclusions

The formation of biofilm within bioreactors causes a reduction in the efficiency of the bioreactor and, in some bioreactor applications, causes health issues. To irradiate biofouling caused problems, good monitoring systems are necessary to develop efficient anti-biofouling strategies. However, the current knowledge does not provide an up-to-date overview of all the different biofilm detection techniques, their pros and cons, monitoring properties, and the different results produced. This study aimed to create an overview of all biofilm detection techniques so that by utilizing this overview, a selection method to choose a biofilm detection technique for specific research can be obtained. To realize this goal, the literature search resulted in 23 different biofilm detection techniques. The primary categorization was based on physical, chemical, microscopical, and biological aspects of applying the alternating biofilm detection techniques. Furthermore, it discusses the biofouling as to whether the methodology applied throughout the research has an influence on the obtained results.

The literature research resulted in a total of 23 biofilm detection techniques. The primary goal of this research was to provide an overview of all biofilm detection techniques. A thorough literature search resulted in these 23 biofilm detection techniques, however, since biofouling is such a wide problem, one cannot be certain of being successful in obtaining all biofilm detection techniques. Furthermore, several biofilm detection techniques such as CUSUM are recent discoveries. This indicates that Table 1 and Table 2 must be continuously adjusted to stay up-to-date. Moreover, many of the stated detection techniques have not yet been applied to bioreactors, and therefore their possible implementation relies completely on the referenced literature. Further research on this topic should be directed toward applying the stated detection techniques for bioreactors so that substantiating literature can be created. Additionally, further research must validate the application of the selection method for other applications. If the application works for other research, it can be stated that the detection tool is properly applicable. 

## Figures and Tables

**Table 1:** Indicative biofilm detection and/or measuring techniques and their advantages and drawbacks based on the literature.

Categ.	Technique	Example of Application	Pros	Cons	Ref.
Physical	Cumulative sum control chart (CUSUM-chart)	Biofilm detection within heat exchangers	Efficient in the early detection of slow and progressive changes within a process Low costs compared to techniques that obtain the same results	Currently measured by the time progression of the heat transfer resistance Rf depends on several other physical-chemical characteristics Experiments are time-consuming	[16,22]
	Visible & Near-Infrared Spectral bands (V&NIR)	Determination of types of biological contaminants existing on the object’s surface	Can be applied outside laboratory (i.e., does not need ideal conditions) Low cost (only costs are photo processing) Versatility (applicable to different species of microorganisms)	Time-consuming A digital camera with the possibility of NIR photos	[15]
	Electrochemical Impedance Method (EIM)	Biofilm detection of surgical implants in the human body	Sensors are highly movable, thus different places in the bioreactor can be measured	Expensive due to sensor costs Difficult interpretation of data due to biofilm heterogeneities	[7,9,29,30,31]
	Nuclear Magnetic Resonance Imaging (NMRI)	Membrane systems in the water industry to produce potable water and for advanced wastewater treatment	Sensitive and can identify biofilm formation at an early stage Does not require object with a homogeneous structure	Time-consuming (large sample preparation time) Many parameters must be determined before starting laboratory experiments	[9,34,35,38,117,118]
	Ultrasonic time-domain reflectometry (UTDR)	Detect biofouling on flat sheet and thin-film membranes in a canary cell configuration	Fast method	Low sensitivity for thin biofilms (i.e., early detection of biofilm formation is less accurate) Special heterogeneity of biofilm makes measurements difficult	[7,9,14,39,41,42,43,44]
	Dry mass weighing (DMW)	Detection and measurements of Candida albicans on dental surfaces	Very easy to perform No need for expensive equipment	Time-consuming Low sensitivity and accuracy when detecting small changes in biofilm production No real-time measurements	[9,47,119]
	Laser-Induced Fluorescence (LIF) spectroscopy	Biofilm detection on the surface of cultural heritage artifacts	Capable of detecting biofilm at an early stage (i.e., slightly after attachment) Chlorophyll fluorescence spectra manifest itself Fast method	Requires expensive measure equipment (e.g., LIF sensors and lasers) Hard to differentiate between bio- and non-biomaterial.	[49,51,53]
	Surface-enhanced Raman spectroscopy (SERS)	Detection of biofouling in drinking water membrane filtration	Differentiating of fouling types and their changes over time (i.e., highly selective) Highly sensitive High speed Minimal requirements for sample preparation	Expensive technique due to the large variety of laboratory equipment needed Special equipment is needed Focusses on a small area (e.g., 30 × 30 μm)	[40,55,56,57]
Chemical	Microtiter plate dye-staining (MPDS) for biomass metabolic activity	Indirect measurement of biofilm metabolic activity by chemical reduction of dye	Versatility High-throughput screening	Lack of reproducibility Lack of sensitivity Wrong estimations are easily made A standardized protocol is not available	[9,62,119,120]
	MPDS for biomass total biomass	Indirect measurement of biofilm biomass by adsorption/desorption of dye (most common dye is CV)	Versatility High-throughput screening	Lack of reproducibility Lack of sensitivity Wrong estimations are easily made A standardized protocol is not available	[9,62,119,121]
	Phospholipid based biomass analysis	Measuring bacterial biomass in sediments	Versatility Good estimation of viability due to rapid degrading of phospholipids in dead cells	Time-consuming Low sensitivity	[9,122]
Microscopical	Light microscopy	Imaging of gram stained section of wound tissue from patients with chronic diabetic foot wounds	Sample preparation cheap and easy to perform Imaging of larger parts of the sample compared to other microscopy detection techniques	Limited magnification and resolution Sample staining necessary Morphotypic differentiation relatively gross Lacking discriminatory detail	[7,9]
	Confocal Laser Scanning Microscopy (CLSM)	Imaging of anti-fouling properties of commercial polymers	Resolution compatible with single-cell visualization Reconstruction of 3D-images of a sample No need for extensive computer processing Applicable for long and short term detection	Usage of expensive fluorophores is necessary There exists interference of the necessary fluorophores and the biofilm properties Destructive technique Obtain a large scan (focus on a small area) Expensive equipment	[14,72,74,86,123]
	Scanning electron microscopy (SEM)	Imaging of bacterial biofilms on steel surfaces	High resolution of images Ability to image complex shapes Wide range of magnifications	Time-consuming sample preparation The sample preparation process can cause sample destruction Destructive technique Unable to obtain a large scan (focus on a small area) Expensive equipment	[9,73,74]
	Atomic force microscopy (AFM)	Imaging of the morphology and mechanical behavior of barnacle cyprid footprint proteins	Works under ambient conditions Same resolution along and perpendicular to the surface Qualitative and quantitative assessment of biofilms	Unable to obtain a large scan (focus on a small area) Expensive equipment	[14,73,83,86]
	Transmission electron microscopy (TEM)	Map the distribution of macromolecular subcomponents of biofilm cells and matrix	Capable of imaging individual microorganisms and their relationship to each other (i.e., high-resolution structural imaging)	Needs specific microscope (e.g., TEM Phillips 300 microscope) Sample needs staining before images can be obtained	[14,85,90]
	Environmental scanning electron microscopy (ESEM)	Demonstration of the degree of exopolymer hydration in manganite-reducing biofilms	Does not require sample preparation (drying, coating) that is required for conventional SEM	Special equipment is needed (i.e., a low vacuum scanning electron microscope) Lower resolution than SEM (10–20 nm)	[71,124]
	Scanning transmission soft X-ray microscopy (STXM)	Map the distribution of macromolecular subcomponents of biofilm cells and matrix	Can be applied to fully hydrated biological materials Provides spatial resolution of <50 nm (i.e., suitable for imaging different bacteria within bacterial biofilms) Allows mapping of chemical species based on bonding structure Minimum sample preparation; sample does not need adding of reflective, absorptive or fluorescent probes	No 3D-imaging Lower resolution than TEM Only relatively thin samples can be visualized (less than 10 microns) Destructive technique; high energy and the high flux of the X-rays causes degradation of the sample shortly after imaging	[14,90]
Biological	Determination of Colony Forming Units (CFU)	Study of the impact of thermal cycling on staphylococcus on orthopedic plates	Easy to perform Can be performed in every microbiology lab	The fraction of detached live cells may not be representative of the initial biofilm population Lack of sensitivity; subpopulation of cells may be viable but non-culturable (VBNC) and will not be detected Limited to microorganisms that develop colonies on agar plates	[12,46,102,103]
	Quantitative polymerase chain reaction (qPCR)	Analysis of the viable bacterial population in a rodent model of dental caries	Fast method (results can be obtained within a few hours) Enables the quantification of different species within one sample	Expensive Inaccurate due to the overestimation of the number of cells due to the presence of DNA	[108,125]
Combination	EPS extraction	Study towards soil biofilm formation and its microbial community diversity and metabolic activity	Possible to, in detail, analyze the composition of EPS	Intercellular content contaminations A microscopy technique, to examine the extracted EPS, must be selected (e.g., SEM & CLSM)	[110]
	Anti-EPS component antibodies	To compare two different vaccines against Staphylococcus Aureus mastitis for sheep	Very high specificity Possible to target a specific component of EPS	Costs are high due to the acquisition of antibodies A microscopy technique must be selected w.r.t. the imaging Antibodies can disturb the signal imaging	[9,126]
	Fourier transform infrared spectroscopy (FTIR-spectroscopy)	Monitoring and detection of biofilm in continuous flow chambers	Requires large calculations Continuously monitored so give a specific time frame of biofilm formation	Many different signals arise from vibrations of molecules in extracellular polymeric substances (EPS) and the cytoplasm. This leads to an overlapping and broadening of bands in the spectra. Expensive due to equipment costs. Long sample preparation time	[73,114,127,128]

**Table 2:** Biofilm detection and/or measuring techniques and their properties and visualization type based on the literature. The different types of detection/monitoring are as follows: in situ, real-time, non-destructive, and online. If one of the references states that the specific technique can perform biofilm detection according to one of these four types of detection/monitoring, this is stated in column 2. Furthermore, two articles may claim different types of detection/monitoring, when this phenomenon occurs, a small description is given within brackets. The third column consists of the monitoring/detection properties of the different techniques. The goal of this column is to summarize the possible results obtained by applying a specific detection technique. Furthermore, the moment of detection is stated (i.e., beginning, middle, or end of biofilm formation phenomena). The fourth column contains a visualization of the result obtained by applying a specific technique. Types of possible results are a graph, picture, or table.

Technique	Type of Detection/Monitoring	Monitoring/Detection Properties	Visualization of Result	Ref.
Cumulative sum control chart (CUSUM-chart)	In situ Real-time Non-destructive Online	Solely the presence of biofilm, this technique is not able to provide any information about the type of biofilm or its structure Detection unknown, whether it is from initial adherence or later	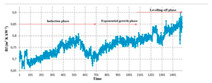	[16,23]
Visible & Near-Infrared Spectral bands (V&NIR)	In situ Non-destructive	Identification of different components of biofilm, not accurately providing information about the structure of the biofilm 2D-distribution of biofilm on the surface (resolution depends on camera) Detection upon the final stage	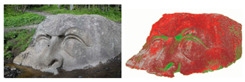	[15]
Electrochemical Impedance Method (EIM)	In situ Real-time Non-destructive Online	Capable of monitoring the presence and amount of biofouling formed on the impedance sensors Detection unknown, whether it is from initial adherence or later	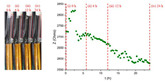	[7,32]
Nuclear Magnetic Resonance (NMR) Imaging	Real-time Online	2D-distribution of biofilm on the surface (max. resolution 220 µm/pixel) 3D-scan (allows visualization and quantification of biofilms and their interaction with the surrounding fluid at mesoscale)	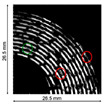	[117,118]
Ultrasonic time-domain reflectometry (UTDR)	In situ Real-time Non-destructive	2D-representation 3D-representation; measure thickness changes Quantitative information about biofouling (c.a. 120 × 10^3^ µm^2^ reflection) Detection upon initial adherence	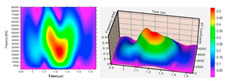	[39,42,44,129]
Dry mass weighing	-	Quantitative information about the biofilm its weight Detection at the final stage	The results gained by weighing and comparing two samples; one clean and the other contaminated by biofouling. The visualization can be in table or graph form or a weight	[46]
Laser-Induced Fluorescence (LIF) spectroscopy	In situ Non-destructive	Capable of providing information about the composition of the biofilm 2D-representation of biofilm on the surface Capable of scanning larger areas and eventually zooming in Detection upon initial adherence	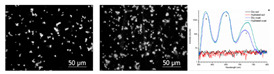	[49,51,53,130]
Surface-enhanced Raman scattering (SERS) spectroscopy	In situ Real-time Non-destructive Online	Capable of differentiating between biofouling types Detection upon initial adherence (single molecular level)	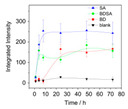	[55,56]
Microtiter plate dye-staining (MPDS) for biomass metabolic activity	In situ (if the biofilm is formed on a microtiter plate)	Capable of determining the presence of biofilm, and if present, the activity of it Detection at the final stage	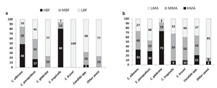	[62]
MPDS for biomass total biomass	In situ (if the biofilm is formed on a microtiter plate)	Capable of determining the total amount of biofilm formed in a given time spectrum Detection at the final stage	The result gained by MPDS for total biomass is comparable to the result gained by MPDS for biomass metabolic activity (i.e., a graph that shows the different percentages of biomass per microorganism)	[62]
Phospholipid based biomass analysis	In situ	Capable of determining the total weight of the biofilm formed Detection upon the final stage	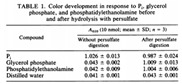	[9,122]
Light microscopy	In situ Non-destructive	2D-distribution of biofilm on the surface (max. resolution 200 nm) Thickness of biofilm Detection upon colonialization (i.e., not capable of imaging at single-cell level)	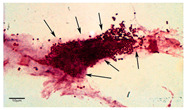	[9]
Confocal Laser Scanning Microscopy (CLSM)	In situ Real-time Non-destructive	2D-distribution of biofilm on the surface (max. resolution 50 µm) 3D-imaging of biofilm and due to this property, capable of obtaining parameters such as biofilm thickness and biofilm roughness Biofilm architecture EPS components participating in the formation of biofilm Organization of microorganisms in the biofilm Detection upon initial adherence	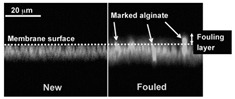	[70,71,78,90]
Scanning electron microscopy (SEM)	-	2D-distribution of biofilm on surface and organization of microorganisms in biofilm (max. resolution 1 nm) 3D-visualization of biofilm and its EPS, capable of obtaining parameters such as biofilm thickness and 3D distribution of microorganisms EPS components participating in the formation of biofilm (and dispersion) Detection upon initial adherence	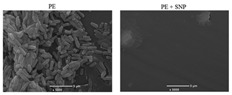	[75,78,79,125]
Atomic force microscopy (AFM)	In situ Non-destructive (not the case when measuring surface thickness)	2D-distribution of biofilm on the surface and surface morphology (max. resolution 1 nm) Capable of measuring surface thickness if part of biofilm is scratched Detection upon initial adherence	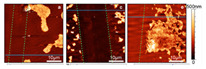	[8,78,87]
Transmission electron microscopy (TEM)	-	2D-distribution of biofilm on the surface (max. resolution 1 nm) Capable of mapping macromolecular composition of the biofilm 3D-associations between nanoparticles of the biofilm & capable of revealing particular structure in EPS Detection upon initial adherence	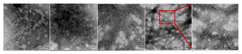	[90]
Environmental scanning electron microscopy (ESEM)	In situ	2D-distribution of large colloid and particle size of biofilm (max. resolution 10 nm) Imaging of the degree of wetting on the surface and inside the biofilm Detection from early stages of colonialization	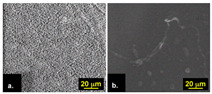	[92,124]
Scanning transmission X-ray microscopy (STXM)	In situ Non-destructive	2D-distribution, localization, and mapping of macromolecules in biofilms (max. resolution <50 nm) Detection from early stages of colonialization	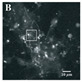	[90,96]
Determination of Colony Forming Units (CFU)	-	Estimates the amount/number of microorganisms present Detection after mature biofilm is transferred to liquid medium (i.e., start of colonization however not in original medium)	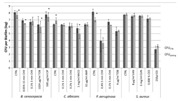	[103]
Quantitative polymerase chain reaction (qPCR)	Real-time	Estimates the amount/number of a specific microorganism and the total amount of present microorganisms Detection from early stages of colonialization	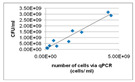	[104,108,131]
EPS extraction	In situ (possibly)	Capable of targeting specific entities of the EPS (e.g., binding of ions in EPS) (ex situ) 3D-structure of biofilms (architecture, distribution, and dynamics during adhesion in biofilm) (in situ + CLSM)	The result will be in the form of an image, however, the type of image depends on the microscopy method applied	[9]
Anti-EPS component antibodies	In situ	Capable of targeting specific components of biofilm, visualization depends on the selected microscopy method	The result will be in the form of an image, however, the type of image depends on the microscopy method applied	[9]
Fourier transform infrared spectroscopy (FTIR-spectroscopy)	In situ Real-time Non-destructive Online	Capable of obtaining chemical information on the different stages of biofilm formation Detection from early stages of colonialization	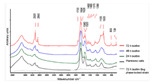	[113,114]

**Table 3:** Factors affecting microbial adhesion to bioreactor surfaces [6,7,14].

Microorganism	Surface	Feedwater
Species	Surface charge	Temperature
Composition of mixed population	Hydrophobicity	pH
Population density	Surface roughness	Dissolved organic matter
Growth phase	Surface topographical configuration (STC)	Dissolved inorganics
Nutrient status	-	Suspended matter
Hydrophobicity	-	Viscosity
Charges	-	Shear forces
Physiological response	-	Boundary layer
-	-	Flux
